# Assessment of the Indoor Odour Impact in a Naturally Ventilated Room

**DOI:** 10.3390/s17040778

**Published:** 2017-04-05

**Authors:** Lidia Eusebio, Marco Derudi, Laura Capelli, Giuseppe Nano, Selena Sironi

**Affiliations:** Politecnico di Milano, Dipartimento di Chimica, Materiali e Ingegneria Chimica “G. Natta”, P.za Leonardo da Vinci 32, 20133 Milano, Italy; lidia.eusebio@polimi.it (L.E.); marco.derudi@polimi.it (M.D.); laura.capelli@polimi.it (L.C.); giuseppe.nano@polimi.it (G.N.)

**Keywords:** odour monitoring, emission factors, indoor air quality, naturally-ventilated rooms, CO_2_, modeling

## Abstract

Indoor air quality influences people’s lives, potentially affecting their health and comfort. Nowadays, ventilation is the only technique commonly used for regulating indoor air quality. CO_2_ is the reference species considered in order to calculate the air exchange rates of indoor environments. Indeed, regarding air quality, the presence of pleasant or unpleasant odours can strongly influence the environmental comfort. In this paper, a case study of indoor air quality monitoring is reported. The indoor field tests were conducted measuring both CO_2_ concentration, using a photoacoustic multi-gas analyzer, and odour trends, using an electronic nose, in order to analyze and compare the information acquired. The indoor air monitoring campaign was run for a period of 20 working days into a university room. The work was focused on the determination of both CO_2_ and odour emission factors (OEF) emitted by the human activity and on the evaluation of the odour impact in a naturally ventilated room. The results highlighted that an air monitoring and recycling system based only on CO_2_ concentration and temperature measurements might be insufficient to ensure a good indoor air quality, whereas its performances could be improved by integrating the existing systems with an electronic nose for odour detection.

## 1. Introduction

Over the last few decades, since the first electronic noses were realized in the 1980s, several studies aimed to investigate some relevant applications of electronic noses for the monitoring of different volatile or semi-volatile pollutants and odour sources.

Nowadays, electronic noses are considered the most promising tools for odour monitoring [1] and the use of these instruments in different sectors has increased [2,3]. In more detail, the research areas in which the application of the electronic noses have been more lively are:

- The food industry, involving several uses, such as process monitoring, freshness evaluation, shelf-life investigation, and authenticity assessment [4,5,6,7,8,9,10]. In Italy, the development of electronic noses for the food industry is very active and focused on freshness evaluation, microbial evaluation and authenticity assessment [11,12,13,14,15,16,17,18,19];
- The healthcare sector that comprehends cosmetics, pharmaceuticals [20,21] and diagnostic [22,23,24,25,26]. In Italy, the research of the electronic nose for the healthcare field is particularly developed in the diagnostic area. Several studies have dealt with issues relating the development of electronic noses as tools for the diagnosis of different cancer types (such as those involving lungs, bladders, and prostates) and the identification of different bacterial infections [27,28,29,30,31,32];
- The environmental sector [33,34,35,36,37,38,39,40]; a recent review [36] on the application of electronic noses in environmental monitoring considered four main research activities: the analysis of parameters related to air quality; the analysis of parameters related to water quality; the process control; and the verification of efficiency of odour control systems. In Italy, the research related to the development and application of the e-nose in the environmental field is very active [41,42,43,44,45,46,47,48,49,50,51,52]. Issues mainly dealt with:
  1. The identification of a method to evaluate the performances of the electronic nose. This topic is particularly interesting internationally because of the recent formation of a technical committee dedicated to the definition of specific regulations of the electronic noses for their standardization;
  2. The analysis of parameters related to air quality;
  3. The analysis of parameters related to water quality;
  4. The process control, as well as the verification of the efficiency of odour control systems.
- The indoor applications [53,54,55,56,57,58,59,60,61] are focused on the detection of the odour compounds that can worsen the air quality and possibly pose some safety problems. Only few Italian studies [62,63,64,65] focus the attention on the indoor air quality (IAQ) monitoring and safety.

The application of the electronic nose in the indoor monitoring, as a tool for the pollution assessment and control, has drawn an increasing interest in the last years. This is confirmed by the increase of the international studies related to the application of these instruments in indoor monitoring [53,54,55,56,59,62,63]. This interest is due to a growing awareness of the indoor air quality of environments where people live and work [64].

The main methods used to monitor and manage indoor environments are generally based on measurements of some physical and chemical parameters [64]. The CO_2_ concentration is often measured and used as a typical marker for the assessment of bad air quality caused by humans [54,55] because both CO_2_ and emissions of bio-effluents (such as some VOCs—Volatile Organic Compounds) are directly related to human metabolism and activities. Comfort criteria, considered in the American Society of Heating, Refrigerating and Air-Conditioning Engineers/American National Standards Institute ANSI/ASHRAE Standard 62-2001 [66], are satisfied if the ventilation of a room guarantees a difference between the indoor and the outdoor CO_2_ concentration below 700 ppm [55]. Furthermore, other standards and guidelines, like the European Standards EN 13779 [67], EN 15251 [68] and the German Standard DIN 1946-2 [69], refer to the CO_2_ concentration to evaluate the ventilation effectiveness and the indoor air quality; these standards advise to keep indoor CO_2_ concentration below 1000 ppm [54,55]. However, it was demonstrated that the monitoring of solely temperature, humidity and CO_2_ concentration could be not exhaustive in order to evaluate the indoor air quality [64]. Indeed, the presence of chemical pollutants and their accumulation with time into indoor environments can cause Building Related Illness (BRI) and Sick Building Syndrome (SBS) disease, especially in buildings with a limited natural ventilation [55] and without a mechanically-controlled ventilation [62].

The indoor air quality is influenced by different factors, such as people’s activities, high occupancy, accidental release of potentially harmful substances or pollutants that come in from external environments, which could change the indoor air composition and affect the living conditions. As it was estimated that about 40% of primary energy in Europe is consumed in buildings to ensure better indoor conditions for occupants, especially for heating, cooling and ventilation [54], a proper IAQ monitoring can lead to an improved life quality decreasing the causes of possible illnesses and defining more efficient ventilation strategies.

Generally, the indoor air quality evaluation is tightly related to olfactory perceptions. Annoying or pungent odours can influence people perception negatively. The standardized method for odour assessment is a sensorial technique called dynamic olfactometry, which uses a human sensory panel. Dynamic olfactometric tests are generally time-consuming and cost-intensive, thus making them not appropriate for a continuous monitoring [54,56]. These disadvantages could be overcome by an instrumental assessment involving the use of an electronic nose.

An overview of relevant international research about the indoor air quality monitoring is summarized in Table 1, which, besides authors and year, reports the application area of the electronic nose and the sensor type adopted.

The following three studies present the Italian research on the indoor air quality monitoring.

The study of Burresi et al. presents a CO commercial sensor for indoor monitoring with the purpose to develop a portable device with both a small size and a low power consumption (around 250 mW) [62].

Zampolli et al. present a work about the environmental safety in indoor monitoring using an electronic nose able to quantify toxic compounds coming from combustion processes (i.e., CO and NO_2_) in spite of the presence of interfering species like VOCs and humidity [63].

Sironi et al. [64] present the preliminary results of an indoor monitoring campaign performed in a naturally ventilated room; in particular, CO_2_ measurements are compared with the results obtained from an electronic nose. This study shows the importance to include the odours monitoring in the procedure to evaluate the indoor air quality. Joining CO_2_ measurements with the information obtained from the electronic nose would allow for optimizing a correct ventilation of the indoor environment.

Nozza et al. [65] present an interesting work on the application of the electronic nose for monitoring the indoor air quality in a room where different kinds of flueless fireplaces were posed. The study presents not only the monitoring of the common pollutants (i.e., CO, CO_2_, NOx, and VOCs) but also the odour emission factors of flueless fireplaces analyzed by separating the contribution of the different operating phases (ignition, shutdown).

The aim of this paper is to present a case study of indoor monitoring in a typical study room naturally ventilated not provided by a mechanical ventilation system. The indoor air monitoring was performed using both an electronic nose to monitor the odours and a photoacustic multi-gas analyzer to detect the CO_2_ over the time for a period of 20 working days. The focus of the work is on the determination of both CO_2_ and odour emission factors (OEF) emitted by the human activity and on the evaluation of the odour impact in a naturally ventilated room. This was done analogously with another study on OEF [65], although the sources analyzed in the present work are multiple and not stationary.

## 2. Materials and Methods

### 2.1. Analytical Equipment

An e-nose for indoor odour monitoring and a photoacoustic multi-gas instrument for the CO_2_ detection were used for the experimental measurements.

**Figure 1:**
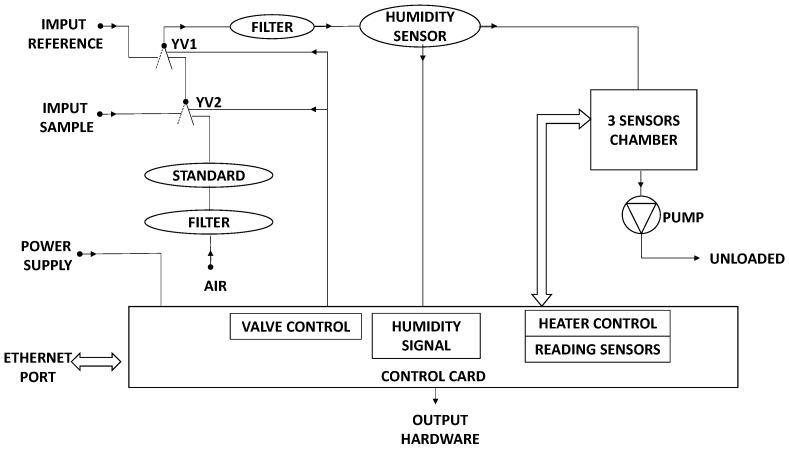
EOS 101 scheme.

The electronic nose used is called EOS 101, provided by Sacmi S.C., Imola, Italy. It is a simplified electronic nose, cheap and small-sized, designed specifically for indoor environments for the determination of the presence/absence of odours; a scheme of EOS 101 is reported in Figure 1.

The EOS 101 is equipped with three Methal Oxide Sensors (MOS) sensors with active film consisting of a Tin oxide thin layer and Tin oxide catalyzed with molybdenum. The sensors are calibrated with a reference substance—n-butanol [65]. The EOS 101 e-nose has two inlets. One inlet is used for the reference air to set the baseline, while the other one to collect the air samples to be analyzed. The reference air was made using a filtration system. The sensor responses are compensated by a software to correct the influence of humidity variations; this is considered enough for indoor applications, characterized generally by low humidity variations.

The EOS 101 has been developed in order to determine the presence/absence of odours, without classifying their quality. This means that the instrument is not designed to classify the analyzed air [64]. EOS 101 continuously analyzes the indoor air and the instantaneous output signal produced by each sensor is expressed in EOS Units (*EU_i_*), which are calculated as follows [65]:

(1) $$\;EU_{i}=a_{i}*{\left(\frac{R_{i}}{R_{std,i}}\right)}^{b_{i}},$$

where *R_i_* is the resistance value, *R_std,i_* is the standard resistance value, and *a_i_* and *b_i_* are characteristic coefficients depending on the sensor type. The so-defined EOS Units can be correlated with the odour concentration (in odour units for cubic meters—ou_E_/m^3^) [65].

The CO_2_ concentration was indeed monitored with an Airnova 1312 photoacoustic multi-gas monitoring system (Limena, Italy). Its measurement principle is based on the photoacoustic infra-red detection method. Some optical filters (up to 5) can be installed in the 1312 filter carousel so that it can selectively measure the concentration of up to 5 gaseous species, such as CO_2_, CO, water vapor, etc. in any air sample. Its detection limit is gas-dependent, but typically is in the ppb region. Reliability of measurement results can be ensured by self-tests that are regularly performed by the instrument. Accuracy is guaranteed by compensating any measurement for temperature fluctuations, water vapor and/or other species interferences.

The gas species detection is ensured by the fact that the light transmitted by the optical filter is selectively absorbed by the gas being monitored, causing the temperature of the gas to increase. Because the light is pulsating, the gas temperature increases and decreases, causing an equivalent increase and decrease in the pressure of the gas (an acoustic signal) in the closed cell. Two microphones mounted in the cell wall measure this acoustic signal, which is directly proportional to the concentration of the monitored gas present in the cell.

### 2.2. Case Study

**Figure 2:**
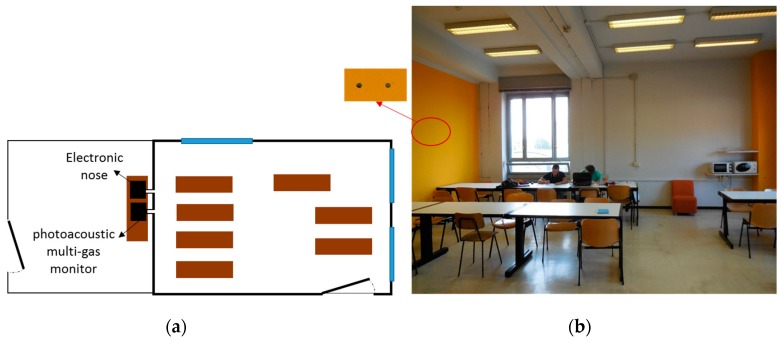
The university study room monitored: Scheme of the study room and the link with monitoring system (**a**); picture of the study room (**b**).

A university study room, not equipped with a mechanical ventilation system, was monitored. Generally, the room taken into account was used for both studying and taking lunch, especially for the time between 12:30 p.m. and 2:30 p.m. (Figure 2).

During the experimental campaign, both the Airnova 1312 photoacoustic multi-gas monitor system and the electronic nose were interfaced with this naturally ventilated chamber thanks to some sampling points placed on the test chamber wall. The instruments were placed in an adjacent room and connected to the study room using a couple of Teflon pipes through sampling holes realized into the wall. From these pipes, air samples of the study room were continuously collected and analyzed both by EOS 101 and the photoacoustic CO_2_ analyzer.

The number of occupants of the investigated room was counted and recorded almost every hour, as well as the activity that was taking place (study and/or lunch) was registered.

The EOS 101 data were processed based on the instantaneous output signal EOS Units (EU_i_) acquired. An average of the EOS Units of the three sensors (EU) was used to calculate specific odour emission factors (OEFs) (ou_E_/h).

With the aim of assessing the characteristic odour impact of the students’ activities, specific odour emission factors (OEFs) (ou_E_/h) were evaluated in terms of odour emitted as a function of time from the room occupants.

In order to determine the OEF relevant to the different activities, an odour material balance on the investigated room was set, according to the following expression:

(2) $$V*\frac{dEU}{dt}=Q*EU_{IN}-Q*EU+OEF,$$

where *V* (m^3^) is the room volume (i.e., 182 m^3^), *Q* (m^3^/h) is the estimated volumetric air flow rate which ensure the natural ventilation of the room, *EU* (that is roughly proportional to an odour concentration, expressed in ou_E_/m^3^) is the average value of the *EU_i_* signals registered by the sensors, and *OEF* is the odour emission factor (ou_E_/h).

Solving the balance, the specific OEF, considered as an average value for people inside the room (*OEF_n_*, expressed in ou_E_/h per person), can be estimated as follows:

(3) $$OEF_{n}=\frac{Q}{n}*\frac{EU_{t}-EU_{t0}*\exp\left[-\frac{Q}{V}*\left(t-t_{0}\right)\right]}{1-\exp\left[-\frac{Q}{V}*\left(t-t_{0}\right)\right]},$$

where *n* is the number of occupants in the investigated room.

Odour Emission Factors (OEFs) are useful tools as they are not only descriptive indicators but also predictive, in agreement with the research performed in an environmental field that brought about the development of OEFs for different kinds of industrial plants such as: wastewater treatment plant (WWTP), Municipal Solid Waste Management Plant (MSWMP), animal rendering plants, and landfills [70,71,72,73,74,75,76,77].

In the case under evaluation, OEFs have been developed in order to assess the odour emissivity due to indoor human activities (i.e., studying and eating). The development of such OEFs is practical as they can be exploited, as an example, in the design of new mechanical ventilation systems for indoor environments.

## 3. Results and Discussion

As previously mentioned, it is useful to properly consider ventilation in order to control the indoor air quality. Among the techniques adopted to perform this kind of evaluations, the one based on the measurement and analysis of the indoor CO_2_ concentration and trends is the most common approach [78,79,80,81]. Even though CO_2_ is usually not considered as a causal factor in human health responses, it can pose some health problems in the case that its concentration level turns out to be very high inside an environment where people may spend a lot of time. ASHRAE standards [66] consider that acceptable ventilation conditions are achieved if an indoor CO_2_ level less than 700 ppm above its outdoor concentration is maintained. While this goal can be easily attained in mechanically ventilated rooms, it is not equally easy to keep CO_2_ and odour levels under control in indoor environments that are naturally ventilated. Natural ventilation is typical of older buildings, such as the one in which the room considered in this study is located, where the ventilation and the IAQ are controlled only by means of the air infiltration through leakages and openings. Into these poorly ventilated and sometimes overcrowded rooms, the situation is even worse during cold seasons, when the desire for thermal comfort and acceptable indoor air quality typically provides conflicting constraints on the ventilation.

To gain some useful information to assess the indoor air quality of the naturally ventilated room previously described, both CO_2_ concentration and odour trends were monitored over time for a period of 20 working days. Experimental results were analyzed and compared with the predictions of typical models used in ventilation studies and for exposure scenario evaluations [65,82,83].

In particular, a simple model can be defined to estimate the indoor air concentration of one or more target species; such a model can be used also to predict the possible dynamics of a ventilation system, of a variation of the generation rate of the pollutants, etc. Starting from the information related to the emission sources and the corresponding emission rates, which in the case of CO_2_ are basically identified by the exhaled breath from the occupants involved in sedentary activities, and with reference to the characteristics of the room, in terms of volume, air exchange rates (AER), etc., a one-box model can be defined to dynamically estimate the indoor CO_2_ concentration trends during the main daily activities as a function of the number of occupants (namely, multiple CO_2_ and odour sources). A constant release rate of CO_2_ was assumed from each point-source; considering that the exhaled breath from each occupant contains about 4% by vol. of CO_2_, this corresponds to about 22 g/h of CO_2_. A well-mixed environment (WMR) was also considered, so the location of the occupants into the room is not relevant, and CO_2_, other possible pollutants and odour should be homogeneously distributed within the room [64,82,83]. Although the investigated room was only naturally ventilated, this simplification could be reasonable because the people movements and their activities improve the mixing between pollutants and indoor air.

Therefore, indoor CO_2_ concentration can be estimated using a single-compartment mass balance model in which a uniform mixing between pollutants and indoor air is assumed:

(4) $$C\left(t\right)=\left(\frac{n\;E}{Q}+C_{in}\right)\left[1-\exp\left(-AER\cdot t\right)\right]+C_{0}\cdot \;\exp\left(-AER\cdot t\right),$$

where *C*(*t*) is the pollutant concentration in the environment (g/m^3^ or mg/m^3^), *C*_0_ the concentration of the pollutant in the room at the beginning of the considered emission period (g/m^3^ or mg/m^3^), *C_in_* the concentration of the pollutant in the air exchanged with the adjacent rooms, *n* the number of occupants, *E* the emission rate (g/h or mg/h), *Q* the air flow rate (m^3^), AER the specific ventilation rate (number of air changes per time unit, defined as the ratio between the ventilation flow rate *Q* and the room volume *V*) (h^−1^), and *t* the time (h).

**Figure 3:**
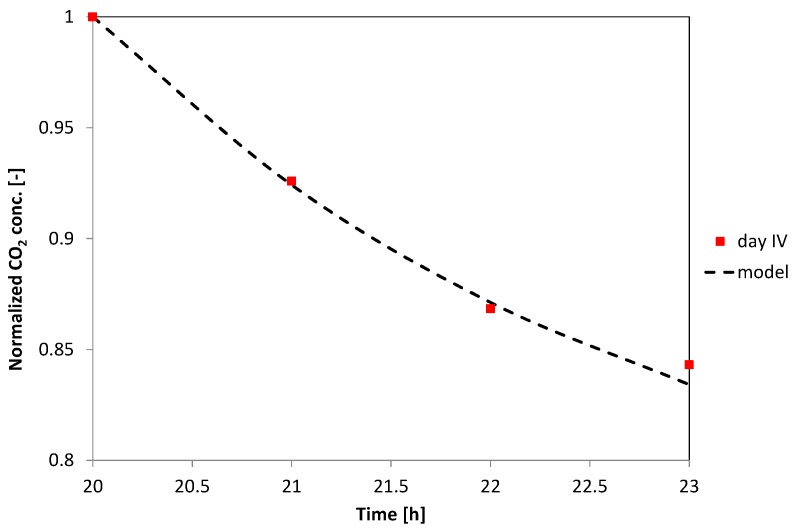
Model fitting of a normalized indoor CO_2_ concentration trend during an unoccupied period for the estimation of the AER (naturally ventilated room).

As the investigated case refers to a naturally ventilated indoor environment, *Q*, and, consequently, *AER* are not known parameters, but they must be estimated. For this reason, the experimental data of the unoccupied periods (from about 8:00 p.m. to midnight) were used to determine the value of *AER*, as shown in the example of Figure 3; the air exchange rate for an unoccupied working period is relatively easier to estimate as the CO_2_ generation rate is zero and the following expression can be used:

(5) $$C\left(t\right)=C_{in}+\left(C_{0}-C_{in}\right)\;\exp\cdot \left(-AER\cdot \;t\right).$$

**Figure 4:**
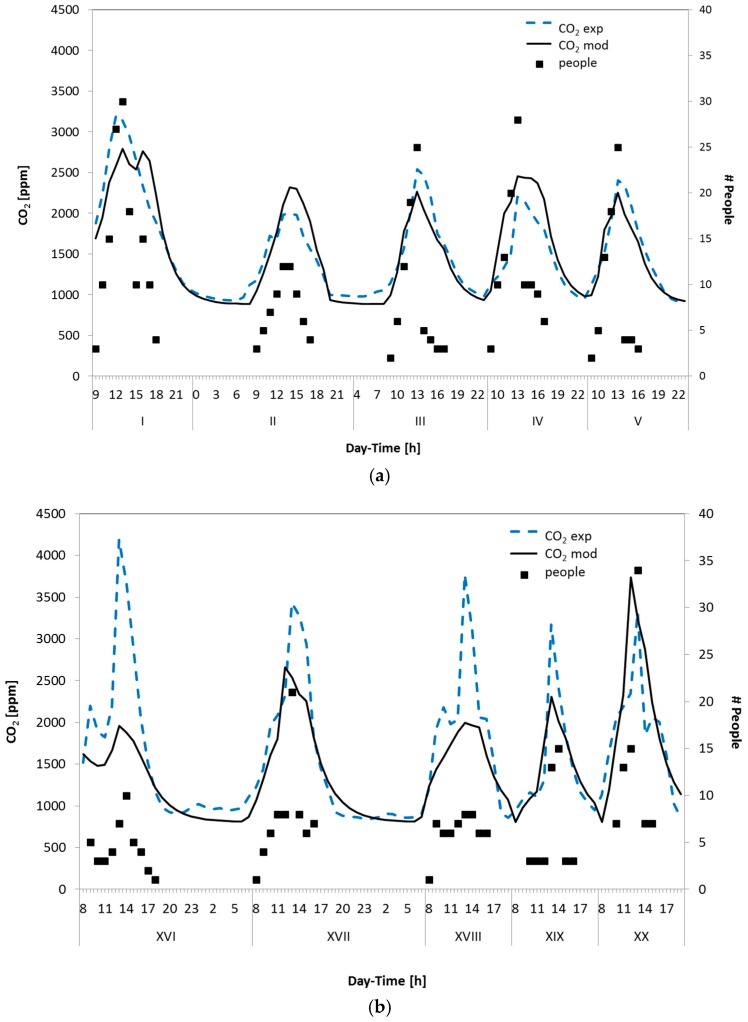
Experimental and predicted CO_2_ concentration trends and number of occupants inside the naturally ventilated room investigated. (**a**) days from I to V; (**b**) days from XVI to XX.

Considering the CO_2_ trends of different days, an average AER value equal to about 0.36 was defined; then, for the occupied periods, when people are present in the room, it was assumed that the daily activities promote both the mixing between air and pollutants and the air exchanges with the external environments; thus, the estimated AER values were increased by a 20% factor. Referring to the number of occupants detected in the study room, the results of the monitoring campaign are evidenced by the black dashed lines of Figure 4. These plots report also a comparison between measured (dashed lines) and predicted (solid lines) CO_2_ trends obtained during two representative working weeks of the experimental campaign. As it can be seen, a reasonable qualitative and quantitative agreement between the CO_2_ measurements and model predictions was obtained.

**Figure 5:**
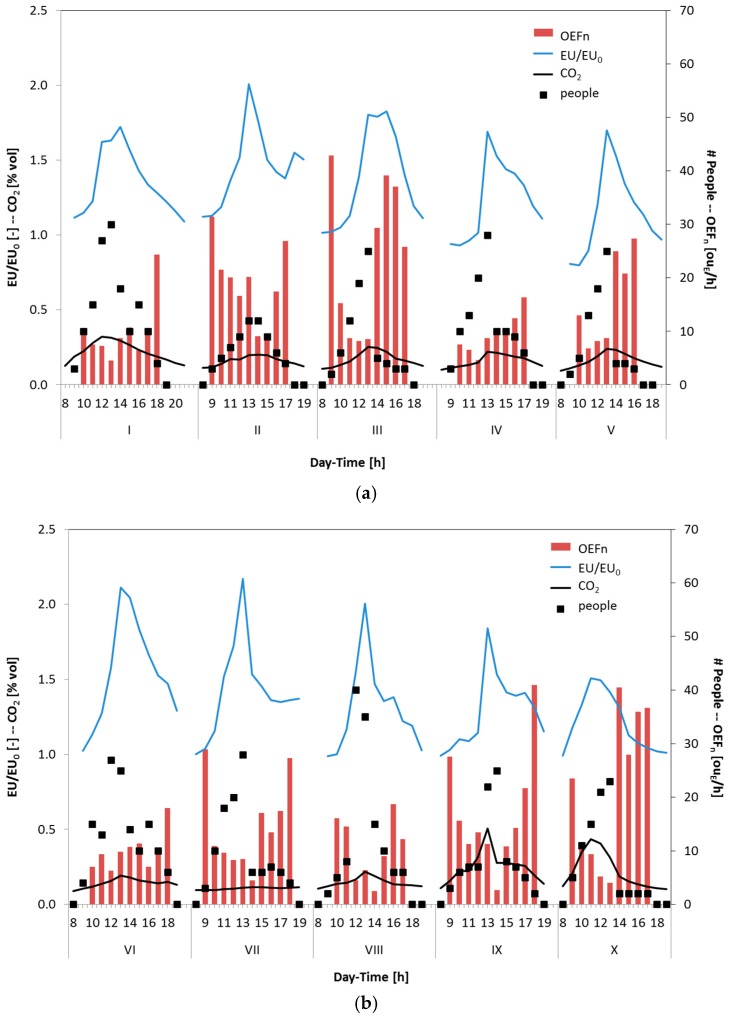
Trends of EU/EU_0_, CO_2_ concentration, number of occupants and estimated OEF_n_ for the period from day I to V (**a**); and for the period from day VI to X (**b**).

**Figure 6:**
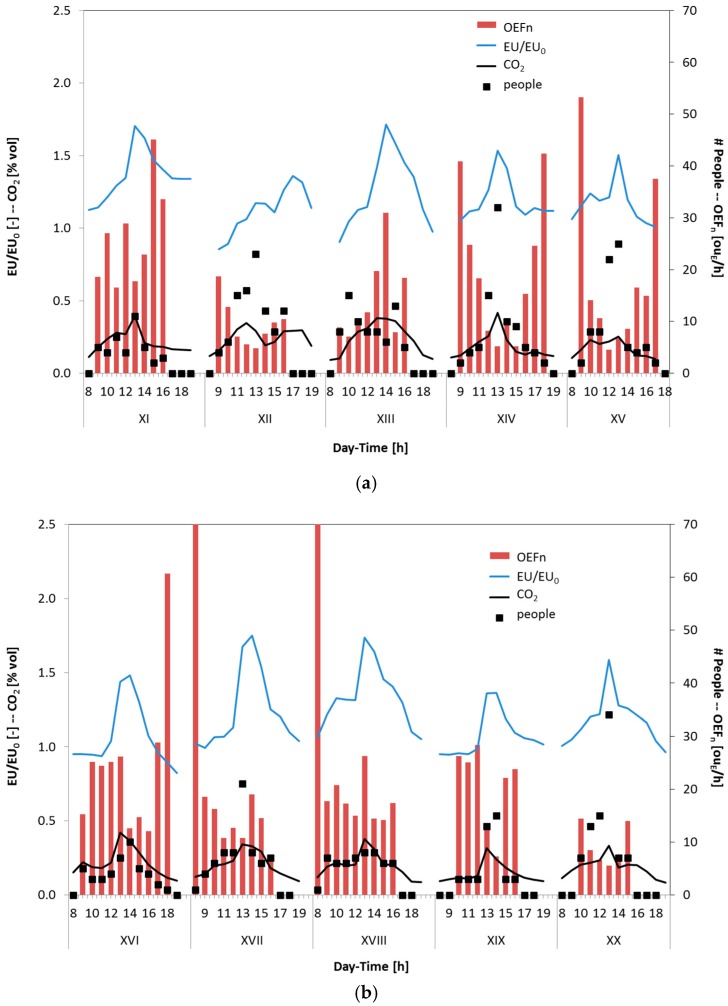
Trends of EU/EU_0_, CO_2_ concentration, number of occupants and estimated OEF_n_ for the period from day XI to XV (**a**); and for the period from day XVI to XX (**b**).

These results confirm that CO_2_ represents a good marker of the human activity in a typical indoor environment; on the other hand, if other possible sources of pollutants and/or volatile chemical compounds are present (such as air fresheners, fireplaces, cooking activities, etc.), monitoring only the CO_2_ trends may not be enough to evaluate in an effective way the indoor air quality. As an example, the odour monitoring and control could be an important issue for air quality evaluation, as evidenced by the experimental results of Figure 5 and Figure 6.

In particular, Figure 5 and Figure 6 show some extracts of the experimental results by reporting the trends of EU/EU_0_, which represent a dimensionless concentration of odour, of the CO_2_ concentration, the people present in the room, as well as the OEF_n_ values derived from the experimental data by means of Equation (3). It is possible to observe several typical rise–decay profiles for both odour (EU/EU_0_) and CO_2_ concentration, with the peaks that occur concomitantly in the range time between 12:00 p.m. and 2:00 p.m., when the room is more populated, and, as a consequence, a higher CO_2_ concentration is expected. Moreover, the principal activity during this time is connected to both studying and eating. Students who gather in the room usually use it to study together and have their meals, releasing a smell that is detected by the e-nose.

It is evident that, on many occasions, the electronic nose and photoacoustic analyzer for the CO_2_ detection provide complementary results. The electronic nose detects odour molecules (i.e., EU/EU_0_) and it is unaffected by variations of CO_2_ concentration, whereas the photoacoustic instrument only detects the CO_2_ and CO concentrations. Although the results underline that both instruments show similar qualitative responses, the two instruments give different information. As an example, it is possible to observe during day VII (Figure 5b) that the CO_2_ concentration measured was low, showing an almost flat profile because the door of the investigated room was left open to improve the air recirculation. The e-nose has nonetheless detected the presence of odours, with trends similar to those recorded in the other days, with an odour peak during lunchtime (12–14).

During the monitored period, it is also possible to observe in some days (i.e., days II, IV, VIII day of Figure 5, and days XIV to XVI of Figure 6) a peak in the EU/EU_0_ signal between 4:00 p.m. and 5:00 p.m. due to the coffee break, whereas the CO_2_ concentration decreases accordingly with the low number of occupants.

In most of the days considered, calculating the average OEF normalized by the number of people present in the room (i.e., OEFn), it is possible to observe an increase of the OEFn values during the late afternoon and the evening when the number of people decreases. This effect cannot be ascribed to the activity carried out by people (i.e., study) but to the presence of other sources and to the accumulation of odour in the room during the day. This can be due to the fact that the molecules of odour generally are larger/heavier than CO_2_ and CO molecules. More in detail, it can be assumed that the odorous emissions produced by the students’ activities are mainly constituted by fatty acids, aldehydes and/or ketones. The molecular weight of these molecules and their steric hindrance, generally, are higher than those of CO_2_ or CO molecules. As a consequence, they require more time to diffuse and to mix with air; thus, a higher time is needed to remove the odour from the room, especially if the air exchange rate cannot be regulated by a mechanical ventilation system. This could be the cause of the high OEFn, often not associated to a corresponding increase of the EU/EU_0_ values, estimated in particular in the post meridian period. This evidence highlights the importance to have forced air changes to better ensure the proper indoor air quality.

In Figure 6b (i.e., days XVII and XVIII), it is possible to observe high OEFn at 8:00 a.m. when the number of people in the room is equal to one. These results cannot be only attributed to the background odour of the room due to furniture but also to the cleaning activity. Indeed, in the morning, between the 7:00 a.m. and 8:30 a.m., the cleaning of the study room was scheduled. The electronic nose has detected the cleaning activity (i.e., OEFn), especially when the cleaning operations are in proximity to the 8:00 a.m., as evidenced in a preliminary study performed by Sironi et al. [64]. Instead, the CO_2_ concentration remains low. The first hours of the day, showing an increase of OEFn due to the cleaning activity, were not taken into account because this aspect was not in the scope of the work.

For comparison purposes, an attempt to extend the modeling approach used for the evaluation of the CO_2_ trends was made to also model odour trends; in this case, the main problems are related to the fact that it is difficult to identify the odour sources and to properly define their emission rates (often not constant) in a complex environment like a study room; moreover, about the odours, there could be some sink effects or secondary reactions that could change the odour distribution within the room because their indoor levels are not solely a function of the sources and the air exchanges.

**Figure 7:**
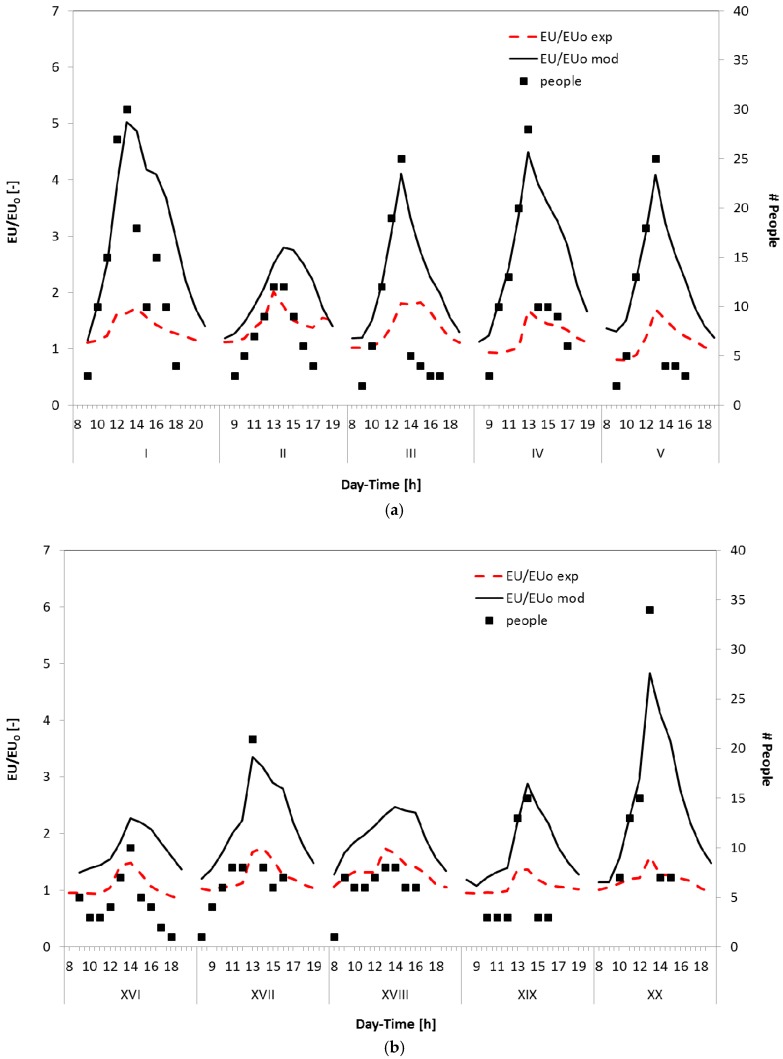
Experimental and predicted EU/EU_0_ trends and number of occupants inside the naturally ventilated room investigated for the period from day I to V (**a**); and for the period from day XVI to XX (**b**).

**Figure 8:**
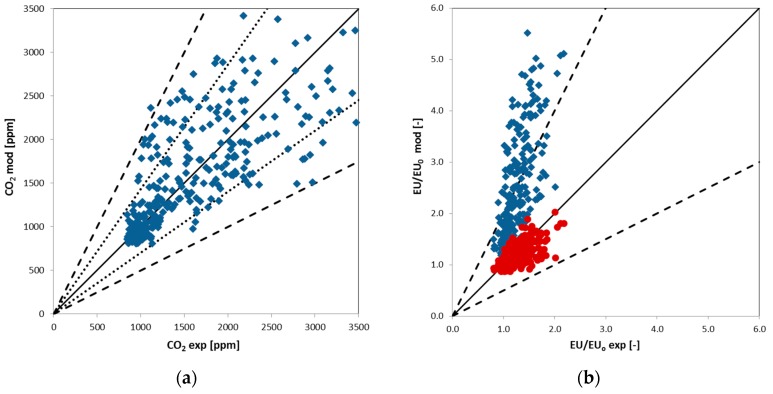
Parity plots of model predictions versus experimental observations for CO_2_ (**a**) and EU/EU_0_ (**b**) found during the indoor monitoring campaign (blue diamonds symbols refer to predictions obtained with average CO_2_ and odour (OEF_n_) emission rates estimated for the room occupants, while red circle symbols represent the predictions obtained considering only a 20% contribution of the room occupants to the odour emission trends).

Therefore, an average value of OEFn was defined on the basis of the specific odour emission factors determined during the experimental campaign; then, considering the number of occupants and the AER previously estimated for the CO_2_ trends, the EU/EU_0_ profiles were predicted for the investigated period; some results are summarized in Figure 7. The model correctly predicts the rise–decay profiles observed experimentally, but there is no quantitative agreement between measured and predicted EU/EU_0_ values. This confirms that, even in a study room, the odour is not related only to people present in this well-defined environment, but a more specific approach is required to correctly identify odour sources and the dynamics of the odour emission trends. As it can be seen from the parity plots of Figure 8, while it is possible to define a reasonable relationship between the occupants and the CO_2_ levels, a more complex situation was evidenced by odour emissions. In particular, in Figure 8, the predicted values of both CO_2_ and EU/EU_0_ were compared with the CO_2_ and EU/EU_0_ values measured during the whole experimental campaign. In these plots, the central solid line represents the ideal values (where experimental results and model predictions fully agree), while dashed lines and dotted lines represent a deviation of ±50% and ±30% from the ideal values. It can be noticed that most of the CO_2_ data are dispersed around the ideal line in the ±30% area; this highlights that the proposed well-mixed room (WMR) model is able to reasonably predict the influence of the room occupants on this pollutant trends; on the other hand, EU/EU_0_ values are clearly overestimated by the model (blue diamonds symbols, right side of Figure 8). The presence of people inside the room surely have an impact on the odour levels, but this contribution seem to have only a small influence on the overall EU/EU_0_ trend. An attempt was made to define the relevance of this contribution and the results are represented by the red circle symbols of Figure 8; this data are well distributed around the ideal line, but, in this case, a reduced OEF_n_ value, equal to 20% of the average OEF_n_ estimated from the experimental results of Figure 5 and Figure 6, was used for the model calculation, thus confirming that other relevant sources, partly independent from the people number, contribute to the definition of the average odour emission rates and, consequently, to determine the measured EU/EU_0_ profiles. This means that odour emissions and trends are due to a complex combination of factors, and a detailed approach is required to control odour levels in indoor environments like the one considered in the present study.

These results show that a monitoring system based only on the CO_2_ measurement might not be enough to properly monitor the indoor air quality, demonstrating that there may be situations where odours can be perceived intensely while the CO_2_ levels remain low. Therefore, an electronic nose system could efficiently integrate the common systems used to regulate the ventilation of indoor environments, in order to allow a better control of indoor air quality.

## 4. Conclusions

The case study reported in this work presents an innovative method to evaluate the indoor air quality. Two different techniques have been combined to monitor a university room: a photoacustic multi-gas analyzer to detect the CO_2_ and an e-nose to detect odour trends. The work focuses the attention not only on CO_2_ concentration and on odour trends (i.e., EU_i_) but also tried to determine the odour emission factors (OEFs) emitted by human activity.

The OEFs are not only descriptive indicators, but they also have a predictive function, in agreement with the research performed in the environmental field that brought about the development of OEFs for different kinds of industrial plants such as waste water treatment plants, animal rendering plants, landfills, etc.

In the present study, OEFs have been developed in order to assess the odour emissivity due to indoor human activities (i.e., studying and eating). The development of such OEFs is of practical interest because they can be used in the design of new mechanical ventilation systems for indoor environments.

The CO_2_ trends evaluated are typical of a naturally ventilated room, where the daily activities promote both the mixing of the air and the pollutants and the air exchanges with the external environments. A reasonable qualitative and quantitative agreement between the CO_2_ measurements and model predictions were obtained using an AER equal to 0.43.

The electronic nose and photoacoustic multi-gas analyzer for the CO_2_ provide complementary information. In some of the days monitored, the e-nose detects odour molecules when the CO_2_ level was low. Moreover, in most of the monitored days, an increase of the odour emissions during the late afternoon and the evening was observed when the number of people decreases. This effect was not due to the activity carried out by the people (i.e., study) but to the accumulation of odour in the room during the day. The molecules of odour generally are larger/heavier than CO_2_ and CO molecules; as a consequence, more time is needed to remove the odour from the room. This evidence highlights the importance of having forced air changes to better ensure the indoor air quality.

These results show that a monitoring system based only on the CO_2_ measurement may not be enough to properly monitor the indoor air quality, demonstrating that there might be situations where odours can be perceived intensely while the CO_2_ levels remain low. Therefore, a small and cheap e-nose system represents an important addition to common systems for the regulation of the mechanical ventilation, based solely on temperature and CO_2_ measurement, in order to allow for an effective control of indoor air quality. Indeed, this study highlights that an e-nose can give supplementary information for the air quality monitoring by detecting odours that are derived from the activities carried out into typical indoor environments.

## Figures and Tables

**Table 1:** International studies focused on the use of e-noses for indoor air quality monitoring.

Authors (Year)	Application Area	Sensor Type ^a^	Reference Number
Arnold et al. (2002)	Use the electronic nose in the indoor environment to detect the early fires	MOS	53
Herberger et al. (2010)	Indoor air quality monitoring using electronic noses to individuate the VOCs produced by human activities and metabolic processes, thus individuating the principal class of compounds that is responsible for odours detected	MOS	54
Herberger and Ulmer (2012)		MOS	60
Kim et al. (2015)		MOS	61
Bitter et al. (2010)	Estimation of odour intensity of indoor air pollutants from building materials	MOS	56
Wolfrum et al. (2006)	Developing of a cheap device for detection, differentiation and quantification of volatile organic compounds at sub-parts-per-million concentration levels	MOS	57
Kuske et al. (2005)	Review about the detection of fungal contamination in indoor environments with electronic nose	CP MOS	58
Tian et al. (2012)	Application of the electronic nose in the monitoring of in-car air quality	MOS	59

Note: ^a^ MOS: metal oxide semiconductors; CP: conducting polymers.
